# A Novel Mutation in *GP1BB* Reveals the Role of the Cytoplasmic Domain of GPIbβ in the Pathophysiology of Bernard-Soulier Syndrome and GPIb-IX Complex Assembly

**DOI:** 10.3390/ijms221910190

**Published:** 2021-09-22

**Authors:** Serena Barozzi, Valeria Bozzi, Daniela De Rocco, Tania Giangregorio, Patrizia Noris, Anna Savoia, Alessandro Pecci

**Affiliations:** 1 Department of Internal Medicine, IRCCS Policlinico San Matteo Foundation and University of Pavia, 27100 Pavia, Italy; serena.barozzi@gmail.com (S.B.); bozzivaleria@gmail.com (V.B.); p.noris@smatteo.pv.it (P.N.); 2 Institute for Maternal and Child Health, IRCCS Burlo Garofolo, 34137 Trieste, Italy; daniela.derocco@burlo.trieste.it (D.D.R.); tania.giangre@gmail.com (T.G.); anna.savoia@burlo.trieste.it (A.S.); 3 Department of Medical Sciences, University of Trieste, 34137 Trieste, Italy

**Keywords:** Bernard-Soulier syndrome, inherited platelet disorders, GPIb-IX complex, vWF receptor, inherited thrombocytopenia

## Abstract

Bernard-Soulier syndrome (BSS) is an autosomal-recessive bleeding disorder caused by biallelic variants in the *GP1BA*, *GP1BB*, and *GP9* genes encoding the subunits GPIbα, GPIbβ, and GPIX of the GPIb-IX complex. Pathogenic variants usually affect the extracellular or transmembrane domains of the receptor subunits. We investigated a family with BSS caused by the homozygous c.528_550del (p.Arg177Serfs*124) variant in *GP1BB*, which is the first mutation ever identified that affects the cytoplasmic domain of GPIbβ. The loss of the intracytoplasmic tail of GPIbβ results in a mild form of BSS, characterized by only a moderate reduction of the GPIb-IX complex expression and mild or absent bleeding tendency. The variant induces a decrease of the total platelet expression of GPIbβ; however, all of the mutant subunit expressed in platelets is correctly assembled into the GPIb-IX complex in the plasma membrane, indicating that the cytoplasmic domain of GPIbβ is not involved in assembly and trafficking of the GPIb-IX receptor. Finally, the c.528_550del mutation exerts a dominant effect and causes mild macrothrombocytopenia in heterozygous individuals, as also demonstrated by the investigation of a second unrelated pedigree. The study of this novel *GP1BB* variant provides new information on pathophysiology of BSS and the assembly mechanisms of the GPIb-IX receptor.

## 1. Introduction

Bernard-Soulier syndrome (BSS; MIM #231200) is a rare autosomal-recessive bleeding disorder caused by biallelic variants in the *GP1BA*, *GP1BB*, and *GP9* genes that encode the subunits GPIbα, GPIbβ, and GPIX, respectively, of the GPIb-IX receptor complex [1]. Homozygous or compound heterozygous mutations induce a loss or severe deficiency of the GPIb-IX complex in the platelet and megakaryocyte membrane [2]. Patients with BSS present with a bleeding tendency, which is usually severe and manifests since the first years of life with mucocutaneous hemorrhages, thrombocytopenia, and platelet macrocytosis with giant platelets [3]. Bleeding manifestations are mainly due to the severe platelet functional defect induced by the GPIb-IX deficiency: in fact, platelet surface expression of GPIb-IX is essential for the initiation of the hemostatic process, as the complex is the platelet receptor for von Willebrand factor (vWF) exposed on the subendothelium at the sites of vascular injury [4]. Thrombocytopenia also contributes to bleeding, though the severity of the bleeding tendency of BSS patients is independent of platelet count [5].

GPIb-IX is a tetrameric receptor complex formed by the association of GPIbα, GPIbβ, and GPIX in a 1:2:1 stoichiometry [4,6]. Each subunit is a transmembrane protein composed of a glycosylated N-terminal extracellular domain, a transmembrane helix, and an intracytoplasmic domain; native proteins include a signal peptide that is removed during protein maturation. The subunits associate in the endoplasmic reticulum and mature in the Golgi apparatus before translocation to the plasma membrane. Expression of the complex on the cell surface depends on the concurrent expression and correct assembly of all three subunits [6,7,8].

To date, at least 120 variants have been identified as responsible for BSS. Pathogenic variants prevent the surface expression of the GPIb-IX complex through different mechanisms, such as the defective expression of the mutated subunit, the impaired association of the subunits into the complex, and/or the defective secretion of the complex to the plasma membrane [2]. The investigation of BSS patients proved to be fundamental not only for elucidating the pathophysiology of the disease, but also for understanding the role of the different domains of GPIbα, GPIbβ, and GPIX in the assembly of the receptor complex on the membrane. Of note, almost all of the reported BSS variants involve the extracellular or transmembrane domains of the three subunits, or, more rarely, the signal peptides [2,4]. The possible role of intracytoplasmic domains is still unclear. To date, only one mutation affecting the intracytoplasmic regions has been identified: The p.Gln587* variant affecting the tail of GPIbα, which causes BSS through a unique mechanism. In fact, homozygous patients have normal levels of the GPIb-IX receptor in the platelet surface, which, however, is unable to bind vWF [9]. Interestingly, some observations on in vitro or animal models have suggested that the alteration of the cytoplasmic domain of the GPIbβ subunit could impair the platelet expression of the vWF receptor and cause a BSS phenotype [10,11]; however, no naturally occurring mutations affecting this region have ever been found in humans to date. 

Herein, we investigated a family with BSS caused by a *GP1BB* variant that induces an almost complete loss of the cytoplasmic domain of GPIbβ. Our findings provide novel information on the pathophysiology of the disease and the mechanisms of GPIb-IX assembly. 

## 2. Results

### 2.1. Clinical Picture of the Family Members

**Figure 1:**
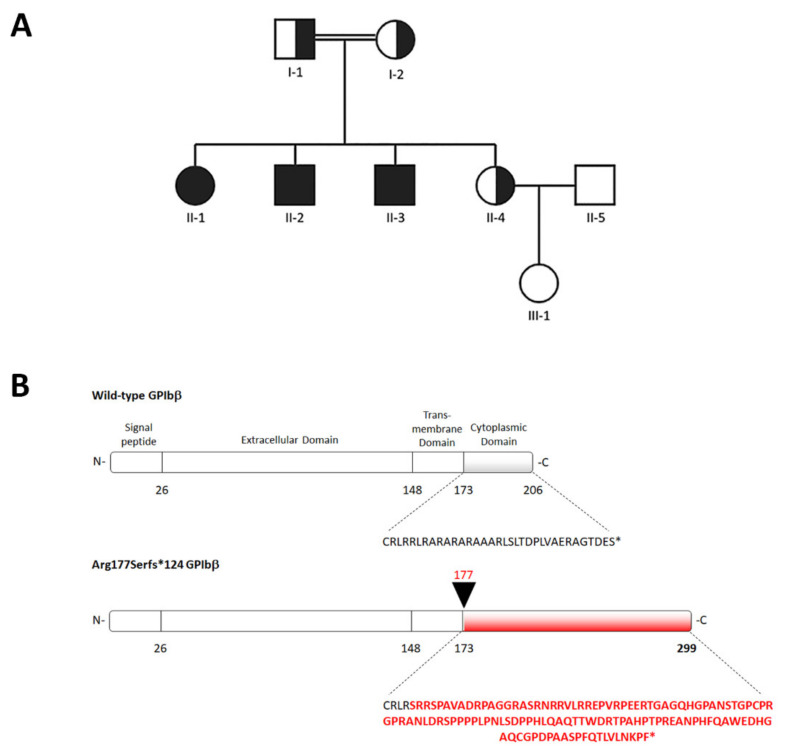
(**A**) Pedigree of the reported family, indicating the analyzed individuals. Black symbols indicate subjects carrying the *GP1BB* c.528_550del variant in homozygosis, and half-black symbols the heterozygous individuals. (**B**) Schematic representation of the sequence of the GPIbβ protein induced by the *GP1BB* c.528_550del variant (p.Arg177Serfs*124). The new amino acid sequence of the tail domain generated by the frameshift variant is indicated in red.

The main clinical features of the family members are summarized in Table 1. The affected individuals were three siblings born to consanguineous parents of Moroccan origin and referred to our institution for investigation of familial thrombocytopenia (Figure 1A). Proband II-1 was a 34-year-old female with a history of thrombocytopenia (platelet count of 14–31 × 10^9^/L) discovered at the age of 32. At this time, after a first diagnostic workup at another hospital, she had received a diagnosis of immune thrombocytopenia (ITP). She was treated with prednisone, then with prednisone plus azathioprine, and subsequently with rituximab, without any increases in platelet count. Treatments for ITP were withdrawn when it became evident that her two younger brothers presented thrombocytopenia too, strongly suggesting a genetic form. At our evaluation, the platelet count was 31 × 10^9^/L with automated counting. Examination of peripheral blood smears revealed prominent platelet macrocytosis with giant platelets (Figure S1). For a more accurate measurement of the platelet count in the presence of marked platelet macrocytosis [12], we performed microscopic counting in a Burker chamber, which showed a platelet count of 56 × 10^9^/L. She reported mild menorrhagia as the only bleeding manifestation; she had previously undergone four dental extractions without any bleeding complications. Patient II-2 was a 33-year-old male presenting with a microscopic platelet count of 45 × 10^9^/L and marked platelet macrocytosis with giant platelets. He did not refer any bleeding symptoms; he previously underwent tonsillectomy and two dental extractions without bleeding complications. Patient II-3 was a 31-year-old male with a hematological picture similar to his older siblings (Table 1). He had been previously treated with prednisone after an initial diagnosis of ITP. He reported no spontaneous hemorrhagic manifestations and two previous dental extractions without bleeding events. Investigation of the other family members revealed that the patients’ father (I-1), as well as their younger sister (II-4), presented mild thrombocytopenia, mild platelet macrocytosis (Figure S1), and no bleeding tendency, whereas their mother (I-2) showed platelet macrocytosis without thrombocytopenia (Table 1). 

### 2.2. Identification of a Novel GP1BB Variant

Next-generation sequencing (NGS) of the proband II-1 identified a homozygous variant, c.528_550del, in the *GP1BB* gene. No other likely pathogenic variants were found in the genes analyzed. Segregation analysis confirmed the variant in homozygosis in the proband II-1 and in her two affected siblings (II-2 and II-3), and found it in heterozygosis in their parents (I-1 and I-2) and younger sister (II-4) (Figure 1A). As shown in Figure 1B, this frameshift variant is predicted to destroy the cytoplasmic domain of GPIbβ starting from the juxtamembrane Arginine 177, and generate an alternative C-terminus that is 93 amino acids longer than the wild-type (p.Arg177Serfs*124). The c.528_550del variant has never been previously reported in patients with thrombocytopenia and is not reported in public databases such as ExAC or GnomAD.

### 2.3. In Vitro Platelet Aggregation

In the three homozygous affected individuals, ristocetin-induced platelet aggregation (RIPA) was reduced after stimulation with 1.5 mg/mL of ristocetin, but presented normal values using a concentration of 3.0 mg/mL (Table S1). Platelet aggregation in response to adenosine diphosphate and collagen was normal (not shown). The three heterozygous individuals (patients’ parents and subject II-4) showed normal platelet aggregation in response to all the tested agonists and concentrations (Table S1 and not shown). 

### 2.4. Flow Cytometry of Platelet Glycoproteins

The results of flow cytometry analysis of the surface glycoproteins of the complexes GPIb-IX and GPIIb-IIIa in the family members are detailed in Table 2. In the homozygous patients, the glycoproteins of the GPIb-IX complex were reduced compared to healthy controls: The mean expression of the complex resulting from the measurement with two different antibodies against GPIbα and one antibody against GPIX was 33.1% of the controls in patient II-1, 31.8% of the controls in II-2, and 29.8% of the controls in II-3. The glycoproteins of the GPIIb-IIIa complex were instead markedly increased, consistent with the prominent platelet macrocytosis of the three patients; in fact, GPIIb and GPIIIa expression was similar between patients and controls after normalization to platelet size (Table S2). The heterozygous individuals showed a mild reduction of the GPIb-IX complex expression: 70.1% and 67.0% of the controls in the two patients’ parents and 67.6% in II-4. In these subjects, GPIIb-IIIa was increased compared to the controls, albeit to a lesser extent than in the homozygous affected individuals, consistent with their milder platelet macrocytosis (Table 2 and Table S2). 

### 2.5. Immunoblotting Analysis of GPIbβ and GPIbα

**Figure 2:**
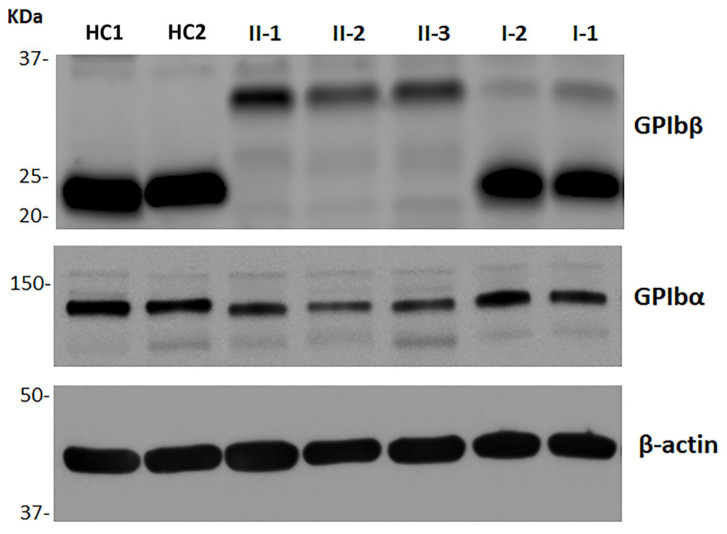
Representative image of the results of the immunoblotting assay for investigation of the total platelet amount of GPIbβ and GPIbα. Whole platelet lysates were obtained from washed platelets of the three patients (II-1, II-2, and II-3), their parents (I-1 and 1-2), and different healthy controls (HCs). β-actin was used as the protein loading control. The results of the densitometric analysis are expressed as the GPIbβ/β-actin and GPIbα/β-actin ratios (see Table 3).

Figure 2 and Table 3 show the results of immunoblotting analysis of whole platelet lysates of the affected individuals and their parents. The c.528_550del variant is expected to induce the synthesis of a GPIbβ larger in size than the wild-type protein (p.Arg177Serfs*124). In fact, in the homozygous patients, the assay for GPIbβ identified a single band at a higher molecular mass (approximately 32 KDa) than that of the wild-type GPIbβ detected in the control subjects (22 KDa). Densitometric analysis showed that the total amount of mutant GPIbβ in the patients’ platelets was 27.1%–29.5% with respect to the healthy controls. The GPIbα content was also reduced, being on average 54.5% in patients compared to controls. In the patients’ parents, the total amount of GPIbβ was only slightly reduced (70.3% and 74.9% of the controls); most of the GPIbβ was constituted by the 22 kDa wild-type form, while only small bands corresponding to the 32 KDa variant could be detected (Figure 2). In heterozygous individuals, the GPIbα content was 77.7% of the controls.

### 2.6. Identification of a Second Family Carrying the c.528_550del Variant

We subsequently identified the c.528_550del variant in *GP1BB* in a second pedigree, which was referred for investigation of autosomal-dominant macrothrombocytopenia. The variant was found in heterozygosis in all the three individuals affected with macrothrombocytopenia (Figure S2). The mutation was identified with NGS and confirmed by Sanger screening; no other variants were found in the analyzed genes. This pedigree was also of Moroccan origin; based on a recounted family history, it was apparently unrelated with the pedigree described above. The main clinical and laboratory features of the affected family members are reported in Table S3.

## 3. Discussion

In this paper, we reported a pedigree carrying a novel variant responsible for BSS, which has the peculiarity of being the first mutation ever identified that affects the intracytoplasmic domain of GPIbβ. In particular, this frameshift variant induces the complete loss of the physiological cytoplasmic domain of the subunit. This investigation of affected individuals provides new information on the pathophysiology of BSS and the assembly mechanisms of the GPIb-IX receptor complex.

Our findings indicate that the loss of the cytoplasmic tail of GPIbβ results in a mild variant of BSS in humans. In fact, the homozygous c.528_550del:p.Arg177Serfs*124 mutation was associated with a 30%–33% expression of the GPIb-IX complex on the platelet surface compared to healthy subjects; this amount appears higher than that reported in most patients with BSS, who usually express less than 10% of the complex [2,5,15,16]. This less severe reduction of the vWF receptor, while inducing the typical macrothrombocytopenia of BSS, resulted in a milder bleeding phenotype. In fact, our patients presented a mild or even absent bleeding tendency and low ISTH BAT bleeding scores, differently from the most common phenotype of BSS patients that is characterized by moderate-to-severe bleeding manifestations and high ISTH BAT scores [5,14,15,17]. Due to the absence of significant bleeding symptoms, thrombocytopenia came to medical attention only in adulthood, thus favoring a misdiagnosis of ITP, as reported in patients II-1 and II-3. The results of the RIPA assay are also in keeping with a mild form of BSS; in fact, our patients showed impaired response to ristocetin 1.5 mg/mL, but normal RIPA using the concentration of 3.0 mg/mL. This response pattern is different from that of all the other BSS patients of our case series, who showed defective RIPA with both ristocetin concentrations [5]. 

The total platelet content of GPIbβ assessed by immunoblotting on whole platelet lysates was 27%–30% compared to healthy subjects, indicating that the p.Arg177Serfs*124 variant results in reduced total GPIbβ expression, most likely due to degradation of the mutant protein, and/or, possibly, instability of the abnormal transcript. Even the total content of the non-mutated GPIbα was significantly reduced in the platelets of our patients; this is consistent with the previous finding that the binding of GPIbβ to GPIbα is essential to prevent the rapid degradation of GPIbα in the lysosome [4,18]. Therefore, if the platelet GPIbβ content is reduced, the platelet GPIbα is also expected to be decreased. 

The expression level of GPIbβ measured by immunoblotting (27%–30% of the controls) was consistent with that of the GPIb-IX complex detected on the platelet membrane by flow cytometry in our patients (30%–33% of the controls). Therefore, the GPIbβ subunit expressed in platelets is correctly assembled into the GPIb-IX receptor on the plasma membrane, indicating that the cytoplasmic domain of GPIbβ is not involved in the assembly and trafficking to the membrane of the GPIb-IX complex. Studies on the organization of the GPIb-IX receptor showed that the association of the transmembrane domains of GPIbα, GPIbβ, and GPIX, and noncovalent interactions between the extracellular domains of GPIbβ and GPIX, are essential for the assembly and translocation of the complex to the cell membrane [4,6,19]. The role of the cytoplasmic domains of the subunits is still unclear, even if the intracytoplasmic tail of GPIbα seems dispensable for the complex expression [9,20,21]. A previous in vitro study on transfected Chinese hamster ovary cells showed that the replacement of the juxtamembrane residues 174–179 of the cytoplasmic domain of GPIbβ with a poly-alanine sequence did not alter the total GPIbβ expression, but completely abolished GPIb-IX assembly, suggesting that this region is essential for trafficking of the complex to the plasma membrane [10]. Our in vivo observations did not confirm this mechanism, since the loss of the cytoplasmic domain of GPIbβ (or, at least, of the residues from the Arginine 177 onward) do not prevent the incorporation in the surface GPIb-IX complex of the total GPIbβ present in platelets. Indeed, the findings in our BSS patients appear strongly consistent with the observations in transgenic mice expressing GPIbβ lacking the intracytoplasmic domain. In fact, these GPIbβΔIC^–/–^ mice, while presenting macrothrombocytopenia typical of the BSS phenotype, expressed approximately 20% of the GPIb-IX complex on the platelet surface and showed a significantly milder bleeding tendency with respect to the GPIbβ^–/–^ knock-out mice, which expressed approximately 3% of the complex and had a severe bleeding phenotype [11].

The intracytoplasmic domain of GPIbβ contains one of the four binding sites of GPIb-IX for the association with the 14-3-3ζ protein, which is involved in the regulation of the affinity of the receptor complex for the vWF; the three other binding sites have been recognized in the cytoplasmic tail of GPIbα [22]. Previous studies have shown that the disruption of this binding site in GPIbβ does not affect interaction of the GPIb-IX complex with 14-3-3ζ, suggesting that this association is maintained through the binding regions in GPIbα [22,23]. Moreover, investigations on both in vitro and animal models demonstrated that the deletion or mutation of the cytoplasmic tail of GPIbβ does not reduce the binding of GPIb-IX to vWF [23,24,25]. Therefore, the loss of the physiologic cytoplasmic domain of GPIbβ induced by the c.528_550del variant is not expected to impair the intrinsic ability of the GPIb-IX receptor to bind to vWF, differently from what happens with the loss of the cytoplasmic domain of GPIbα [9,24]. Consistently, the mild bleeding phenotype of our BSS patients and the results of the RIPA assay do not suggest the eventuality of a defective function of the GPIb-IX receptor in addition to its reduced expression on the platelet surface.

Heterozygous carriers of BSS mutations are usually not expected to have platelet alterations. However, a limited number of variants in the *GP1BA* or *GP1BB* genes have been previously associated with mild autosomal-dominant macrothrombocytopenia; of note, most of these variants have been implicated in classical biallelic BSS when present in homozygosis or compound heterozygosis [2,26,27]. Our findings indicate that even the c.528_550del variant in *GP1BB* exerts a dominant effect. In fact, the heterozygous individuals I-1 and II-4 of our family presented macrothrombocytopenia, while the subject I-2 showed only platelet macrocytosis. Moreover, our conclusion is strongly supported by the identification of a second unrelated pedigree in which the same variant in heterozygosis segregates with autosomal-dominant macrothrombocytopenia. Overall, in the five heterozygous individuals with thrombocytopenia, the reduction in platelet count was mild to moderate; in all of the six heterozygous subjects, the degree of platelet macrocytosis was lower than that of the homozygous patients. The heterozygous patients did not report bleeding symptoms. Whenever investigated, platelet surface expression of the GPIb-IX complex was approximately 70% of controls and the total platelet content of GPIbβ was reduced to a similar extent. Consistent with previous observations [27,28], RIPA was normal. Thus, our results enlarge the spectrum of *GP1BB* variants associated with autosomal-dominant macrothrombocytopenia without a significant bleeding tendency and suggest a critical role for the cytoplasmic tail of GPIbβ in platelet production.

In summary, the loss of the cytoplasmic domain of GPIbβ results in a mild form of BSS. This phenotype is associated with a reduced total platelet expression of the mutant subunit, which is, however, correctly assembled into the GPIb-IX complex on the platelet surface. 

## 4. Patients and Methods

### 4.1. Patients

Investigation of the family members was performed at the IRCCS Policlinico San Matteo Foundation, Pavia, Italy. This study was approved by the Institutional Review Board of Pavia. All investigated individuals or their legal guardians provided written informed consent for the study, which was conducted in accordance with the Declaration of Helsinki.

### 4.2. Platelet Aggregation

Platelet aggregation was studied according to the densitometric method of Born, as reported [29]. The following agonists and concentrations were used: Collagen (4 µg/mL) (Mascia Brunelli, Milan, Italy), adenosine diphosphate (5 µM) (Sigma-Aldrich, St Louis, MO, USA), and ristocetin (1.5 and 3.0 mg/mL) (Sigma-Aldrich).

### 4.3. Flow Cytometry of Platelet Surface Glycoproteins

The surface expression of platelet glycoproteins was investigated by flow cytometry, as reported [30]. The following FITC-conjugated monoclonal antibodies from Immunotech (Marseille, France) were used: P2 against GPIIb in the intact complex with GPIIIa (CD41); SZ2 against GPIbα (CD42b); MB45 against GPIbα (CD42b); SZ1 recognizing GPIX (CD42a) when correctly complexed with GPIbα; mouse IgG1 isotype control. FITC-conjugated VIPL2 against glycoprotein GPIIIa (CD61) was from Immunostep (Salamanca, Spain). P2 against CD41 was used to gate platelets. The data obtained from each investigated subject are expressed as the percentages of the mean fluorescence intensity compared to four healthy controls processed in parallel. 

### 4.4. Immunoblotting Assay

The general procedures of immunoblotting analysis of whole platelet lysates have been previously described [30,31]. Briefly, lysates of washed resting platelets were dissociated under reducing conditions, loaded on gradient 4%–20% gels, and transferred to nitrocellulose (Biorad, Hercules, CA, USA). Membranes were probed with the following antibodies: Rabbit polyclonal against GPIbβ (Novus Biological, Littleton, CO, USA); mouse SZ2 against GPIbα (Immunotech); mouse AC-15 against β-actin (Sigma-Aldrich). The appropriate HRP-conjugated secondary antibodies (Dako, Santa Clara, CA, USA) were used for detection. The patient’s samples were processed in parallel with those of four healthy controls (the same healthy individuals used as controls for the flow cytometry study). Densitometric analysis of the bands was performed by the ImageJ software (National Institutes of Health, Bethesda, MD, USA); data are expressed as the percentages with respect to healthy individuals and represent the means ± SD of two separate experiments.

### 4.5. Mutation Screening

For mutational genetic screening, we used the NGS Ion PGM^TM^ platform (IPGM; Life Technologies, Waltham, MA, USA) to sequence the coding and intronic flanking regions of genes responsible for different forms of inherited thrombocytopenias, as reported [32,33]. The genes analyzed were *RBM8A* (OMIM 605313), *MPL* (OMIM 159530), *HOXA11* (OMIM 142958), *RUNX1* (OMIM 151385), *ANKRD26* (OMIM 610855), *FLI1* (OMIM 193067), *GATA1* (OMIM 305371), *NBEAL2* (OMIM 614169), *MYH9* (OMIM 160775), *WAS* (OMIM 300392), *ACTN1* (OMIM 102575), *FLNA* (OMIM 300017), *GP1BA* (OMIM 606672), *GP1BB* (OMIM 138720), *GP9* (OMIM 173515), *VWF* (OMIM 613160), *ITGA2B* (OMIM 607759), *ITGB3* (173470), *TUBB1* (612901), *CYCS* (123970), *ABCG5* (OMIM 605459), and *ABCG8* (OMIM 605460). The variants were confirmed and analyzed in family members by Sanger sequencing.

## Figures and Tables

**Table 1:** Main clinical and laboratory features of the members of the reported family.

Subject	Gender/ Age (Years)	Automated Platelet Count, × 10^9^/L ^1^	Microscopic Platelet Count, × 10^9^/L ^2^	MPV, Fl ^3^	Mean Platelet Diameter, µm ^4^	Giant Platelets ^5^	ISTH BAT Score ^6^	Bleeding Symptoms
I-1	M/59	107	129	14.1	3.07	No	0	None
I-2	F/55	175	197	14.5	2.95	No	0	None
II-1	F/34	31	56	20.4	4.05	Yes	1	Mild menorrhagia
II-2	M/33	22	45	18.9	4.45	Yes	0	None
II-3	M/31	17	55	20.2	4.61	Yes	0	None
II-4	F/30	103	110	14.5	3.01	No	0	None

Notes: ^1^ Evaluated by automated cell counter, reference values 150–400 × 10^9^/L. ^2^ As determined by phase contrast microscopy in a counting chamber, reference values 150–400 × 10^9^/L. ^3^ Mean platelet volume (MPV) evaluated by automated cell counter, reference values 8–13 fL. ^4^ Evaluated on blood smears by software-assisted image analysis, as previously reported [13]. Reference value obtained from 55 investigated healthy volunteers was 2.58 µm with 95% CI of 2.4–2.7. ^5^ Platelets larger than a red blood cell (8 µm) upon microscopic examination of blood smears. ^6^ The International Society on Thrombosis and Haemostasis (ISTH) Bleeding Assessment Tool (BAT) score was assessed as previously reported [14].

**Table 2:** Flow cytometry analysis of platelet surface glycoproteins.

Subject	GPIbα (SZ2), % of Controls	GPIbα (MB45), % of Controls	GPIb-IX (SZ1), % of Controls	GPIIb (P2) % of Controls	GPIIIa (VIPL2), % of Controls
II-1	36.7 ± 2.1	31.1 ± 2.2	31.5 ± 2.9	231.8 ± 3.1	205.2 ± 4.3
II-2	37.2 ± 1.1	29.3 ± 1.4	28.9 ± 1.3	252.2 ± 14.4	215.3 ± 10.2
II-3	29.7 ± 3.2	31.4 ± 1.5	28.5 ± 1.8	267.3 ± 8.7	199.5 ± 12
I-1	62.8 ± 2.9	87.3 ± 3.9	60.2 ± 4.3	155.6 ± 10.4	161.7 ± 14.2
I-2	63.7 ± 2.5	69.7 ± 5.4	67.7 ± 5.6	139.0 ± 8.5	145.3 ± 7.3
II-4	70.1 ± 6.9	65.3 ± 4.3	67.3 ± 3.7	145.5 ± 3.8	155.3 ± 6.9

Note: Expression of the glycoproteins was calculated as the percentages of the mean fluorescence intensity with respect to healthy individuals processed in parallel (controls) and represent the means ± SD of two separate experiments.

**Table 3:** Densitometric analysis of the GPIbβ and GPIbα bands obtained with immunoblotting assay.

Scheme	GPIbβ, % of Controls		GPIbα, % of Controls
	22 kDa	32 kDa	
II-1	0	27.1 ± 0.3	58.4 ± 7.1
II-2	0	27.1 ± 2.6	49.9 ± 7.5
II-3	0	29.7 ± 2.5	55.2 ± 6.3
I-1	67.4 ± 7.5	7.5 ± 1.7	73.5 ± 9.5
I-2	66.9 ± 3.9	3.4 ± 0.5	82.0 ± 9.1

Note: The glycoprotein amounts in whole platelet lysates were calculated as the GPIbβ/β-actin and GPIbα/β-actin ratios, respectively. Data are expressed as the percentage with respect to healthy individuals processed in parallel (controls) and represent the means ± SD of two separate experiments.

## Data Availability

The data presented in this study are available in the main text and the Supplementary Materials.

## Supplementary Materials

The following are available online at https://www.mdpi.com/article/10.3390/ijms221910190/s1.
