# Supplementary material for: A Novel Mutation in GP1BB Reveals the Role of the Cytoplasmic Domain of GPIbβ in the Pathophysiology of Bernard-Soulier Syndrome and GPIb-IX Complex Assembly
Source: Int J Mol Sci. 2021 Sep 22;22(19):10190. doi: 10.3390/ijms221910190 (PMC8508601; doi:10.3390/ijms221910190)
Supplement: Supplementary file 1 [file ijms-22-10190-s001.zip › ijms-1367598-supplementary.pdf]

Supplementary materials of the paper entitled "A novel mutation in *GP1BB* reveals the role of the cytoplasmic domain of GPIbβ in the pathophysiology of Bernard-Soulier syndrome and GPIb-IX complex assembly" by Barozzi et al.

SUPPLEMENTARY TABLES

Table S1. Results of the ristocetin-induced platelet aggregation (RIPA) assay.

Subject	RIPA, % of controls	
	**1.5 mg/mL**	**3.0 mg/mL**
II-1	18	81
II-2	15	75
II-3	21	83
I-1	77	nd
I-2	85	nd
II-4	85	nd

Note: Data are expressed as the percentage with respect to healthy individuals processed in parallel (controls): normal range for our laboratory is 67-90% for ristocetin 1.5 mg/mL and 70-100% for ristocetin 3.0 mg/mL.
Abbreviations: nd = not determined.

Table S2. Results of flow cytometry analysis of platelet surface glycoproteins after normalization to platelet size.

Subject	GPIbα (SZ2), % of controls	GPIbα (MB45), % of controls	GPIb-IX (SZ1), % of controls	GPIIb (P2) % of controls	GPIIIa (VIPL2), % of controls
II-1	17.9 ± 2.7	15 ± 0.4	15.4 ± 2.9	107.6 ± 12.4	99.5 ± 7.5
II-2	20.11 ± 6.9	15.6 ± 2.7	15.2 ± 0.2	112.4 ± 5.1	113.4 ± 12.6
II-3	15.5 ± 3.1	16.2 ± 0.8	14.8 ± 0.5	118.3 ± 8.8	103.7 ± 16.3
I-1	39.6 ± 5.4	54.7 ± 2.8	37.6 ± 0.9	97.5 ± 2.7	101.9 ± 18.4
I-2	44.7 ± 2.5	48.8 ± 0.8	47.5 ± 0.7	97.4 ± 3.5	102.4 ± 14.9
II-4	49.2 ± 9.5	45.7 ± 7.4	47.1 ± 7.1	101.2 ± 6.5	108.7 ± 15.4

Note: the mean fluorescence intensity values obtained with each antibody (Table 2) were normalized to forward scatter channel data, and expressed as the percentages with respect to healthy individuals processed in parallel (controls); data represent the means ± SD of two separate experiments.

Table S3. Main clinical and laboratory features of the members of a second family presenting autosomal-dominant macrothrombocytopenia that segregates with the *GP1BB* c.528_550del variant in heterozygosis (see Figure S2).

Subject	Gender/ age (years)	Automated platelet count, $x10^9/L$[1]	Microscopic platelet count, $x10^9/L$[2]	MPV, fL[3]	Mean platelet diameter, µm[4]	Giant platelets[5]	ISTH BAT score[6]	Bleeding symptoms
I-1	M/67	111	131	13.1	3.2	no	0	none
II-1	F/44	94	107	13.5	3.2	no	0	none
II-2	F/42	54	68	14.2	3.6	no	0	none

Notes: [1] = evaluated by automated cell counter, reference values 150-400 $x10^9/L$. [2] = as determined by phase contrast microscopy in a counting chamber, reference values 150-400 $x10^9/L$. [3] = Mean Platelet Volume (MPV) evaluated by automated cell counter, reference values 8-13 fL. [4] = evaluated on blood smears by software-assisted image analysis, as previously reported [13]. Reference value obtained in 55 investigated healthy volunteers was 2.58 µm with 95% CI 2.4–2.7. [5] = platelets larger than a red blood cell (8 µm) at microscopic examination of blood smears. [6] = the International Society on Thrombosis and Haemostasis (ISTH) Bleeding Assessment Tool (BAT) score was assessed as previously reported [14].

SUPPLEMENTARY FIGURES

Figure S1

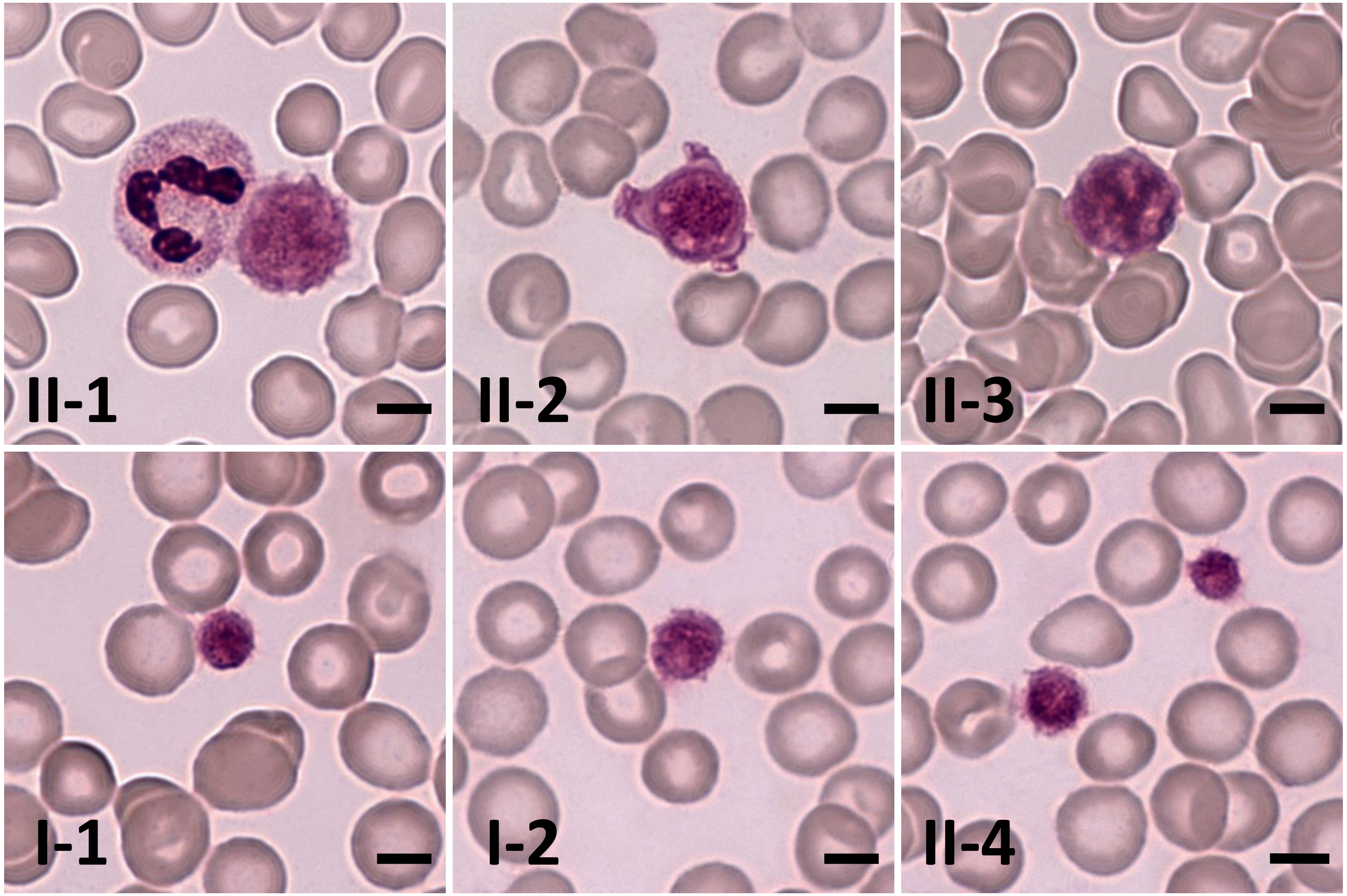

FIGURE S1. Representative examples of platelets of family members detected on examination of peripheral blood smears (May-Grünwald-Giemsa staining). Patients II-1, II-2, and II-3, who carried the c.528_550del variant in homozygosis (Figure 1A), presented marked platelet macrocytosis with giant platelets (platelets larger than red blood cells). Heterozygous individuals I-1, I-2, and II-4 also showed platelet macrocytosis, albeit to a lesser degree than homozygotes. Scale bars correspond to 5 µm. Mean platelet diameter in healthy individuals is 2.58 µm with 95% CI 2.4–2.7 [13].

Figure S2

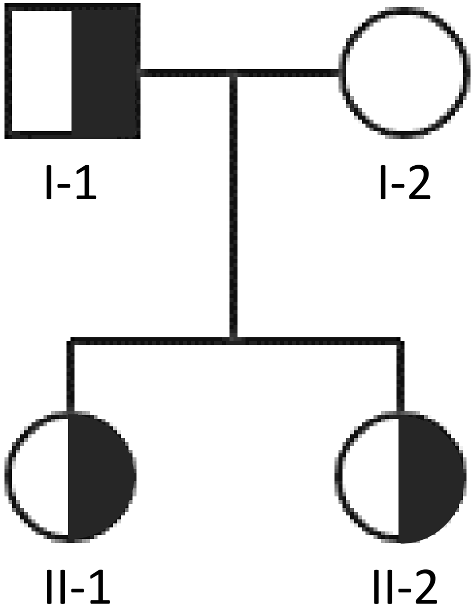

FIGURE S2. Pedigree of a second family of Moroccan origin carrying the *GP1BB* c.528_550del variant. Individuals I-1, II-1 and II-2 presented with autosomal-dominant macrothrombocytopenia (Table S3); genetic analysis identified the *GP1BB* c.528_550del variant in heterozygosis in all the affected subjects (half-black symbols).
